# Supplementary figures and images for: The RNA export and RNA decay complexes THO and TRAMP prevent transcription-replication conflicts, DNA breaks, and CAG repeat contractions
Source: PLoS Biol. 2022 Dec 27;20(12):e3001940. doi: 10.1371/journal.pbio.3001940 (PMC9829180; doi:10.1371/journal.pbio.3001940)

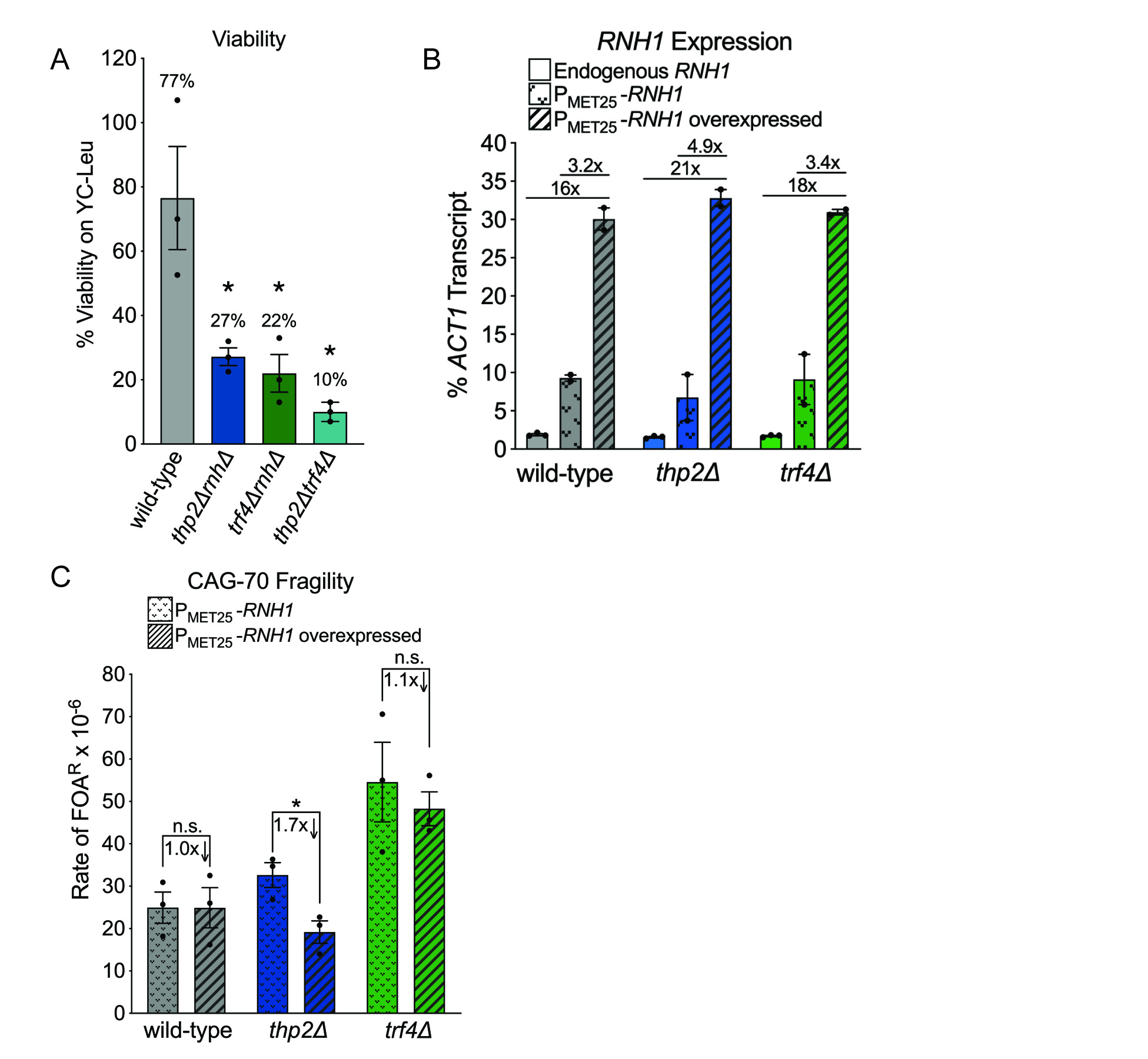

Supplement: S1 Fig — (A) Frequencies of viability are shown. Viability is calculated by comparing the amount of cells that grew into colonies on YC-Leu plates to the amount of cells plated, counted by hemacytometer. Each data point represents an individual biological replicate; average ± SEM is shown; *p < 0.05, compared to wild-type, t test (Table F in S1 Tables). (B) RNH1 transcript levels in 3 conditions: the native RNH1 gene under its own promoter, the RNH1 gene placed under the MET25 promoter (PMET25-RNH1) uninduced, and PMET25-RNH1 induced in media lacking methionine and cysteine. mRNA was reversed transcribed into cDNA by RT-PCR and qPCR was used to quantify cDNA at RNH1 and ACT1 gene loci. RNH1 qPCR signal was normalized to the ACT1 qPCR signal in the indicated mutants (Table I in S1 Tables). (C) Rate of FOAR × 10−6 in indicated strains containing PMET25-RNH1 grown with methionine (no Rnh1 induction) or without methionine (Rnh1 induced); each data point represents an individual biological replicate of a 10-colony assay; *p < 0.05, compared to no induction condition in the same mutant, by t test. Average of at least 3 experiments ± SEM is shown (Table A in S1 Tables). Note that even under non-induced conditions (+ methionine), the RNH1 gene is slightly overexpressed when under control of the non-native MET25 promoter and the fragility rates are higher than when RNH1 is expressed under its native promoter, especially for the wild-type strain (compare rate to Fig 1B). (TIF) [file pbio.3001940.s001.tif]

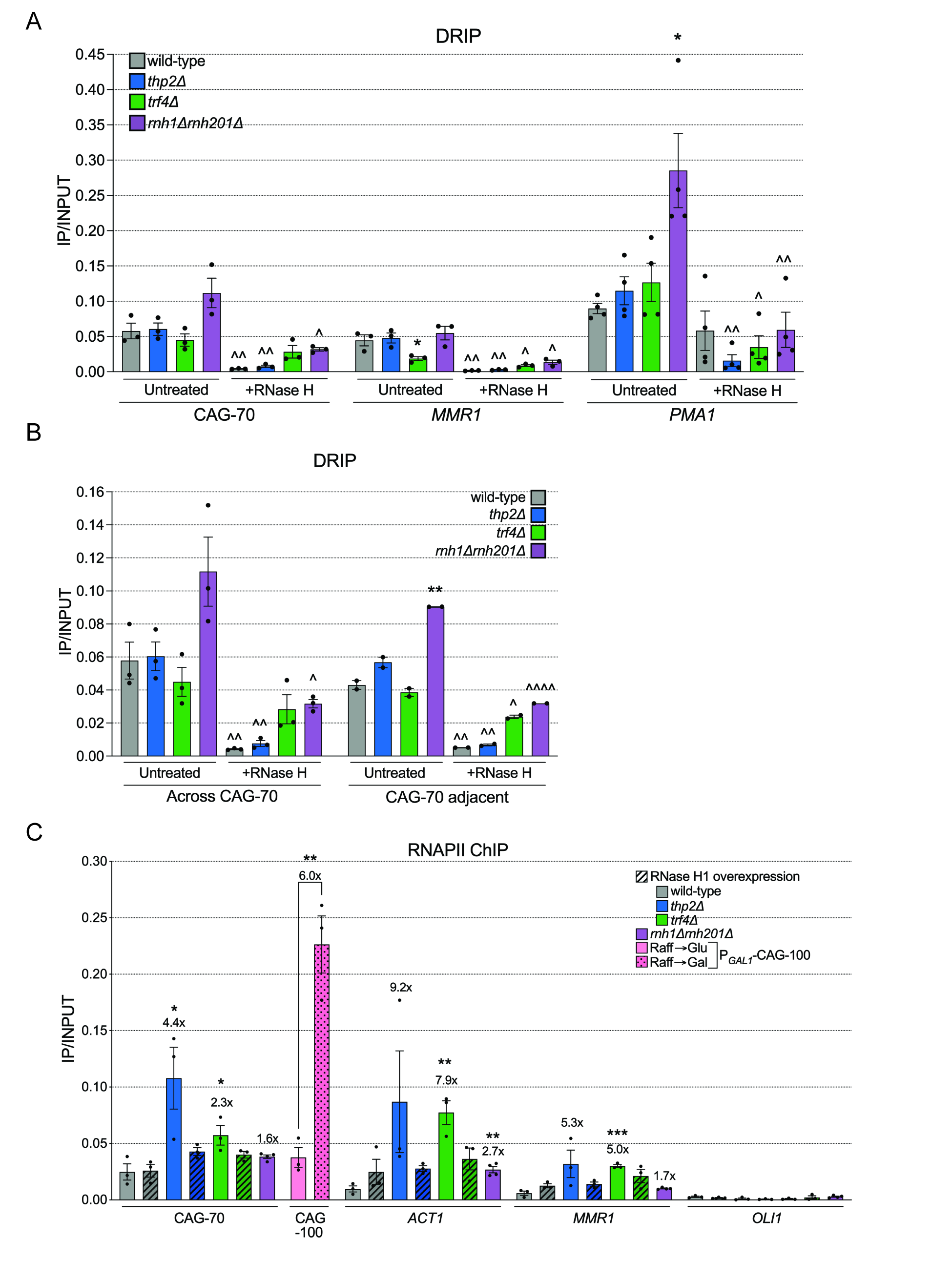

Supplement: S2 Fig — (A) DRIP-qPCR shown as IP/INPUT in wild-type, thp2Δ, trf4Δ, and rnh1Δrnh201Δ strains with and without RNase H treatment. Each bar represents the mean ± SEM of at least 3 biological replicates; each data point represents an individual biological replicate. PMA1 and MMR1 loci were evaluated as loci previously shown to have high and low R-loops, respectively [27,94], as a comparison to the CAG locus. *p < 0.05 compared to wild-type, ^p < 0.05 and ^^p < 0.01 compared to corresponding strain with no treatment, by t test (Table G in S1 Tables). (B) DRIP-qPCR comparing results using primer sets that either span the CAG-70 tract (across-CAG, 345 bp) or are directly adjacent to the CAG-70 tract (CAG adjacent, 101 bp; see Fig 3A) in wild-type, thp2Δ, trf4Δ, and rnh1Δrnh201Δ strains with and without RNase H treatment. Each bar represents the mean ± SEM of at least 2 biological replicates; each data point represents an individual biological replicate. *p < 0.05 compared to wild-type, ^p < 0.05, ^^p < 0.01, and ^^^^p < 0.0001 compared to corresponding strain with no treatment, by t test (Table G in S1 Tables). (C) RNAPII ChIP shown as IP/INPUT in the indicated strains either without (RNH1 endogenous promoter) or with RNase H1 overexpression (RNH1 expressed under the PMET25 promoter, induced in the absence of methionine). A wild-type strain with a galactose inducible promoter driving transcription through a CAG-100 repeat tract was done as a positive control (cells are grown in raffinose-containing media and then split into glucose and galactose-containing media; growth in glucose is no induction, growth in galactose is induction of transcription). The OLI1 locus was included as a negative control since OLI1 is in the mitochondrial genome and is not transcribed by RNAPII. Each bar represents the mean ± SEM of at least 3 biological replicates; each data point represents an individual biological replicate. *p < 0.05, **p < 0.01, ***p < 0.001 compared to wild-type or strain indicate [file pbio.3001940.s002.tif]

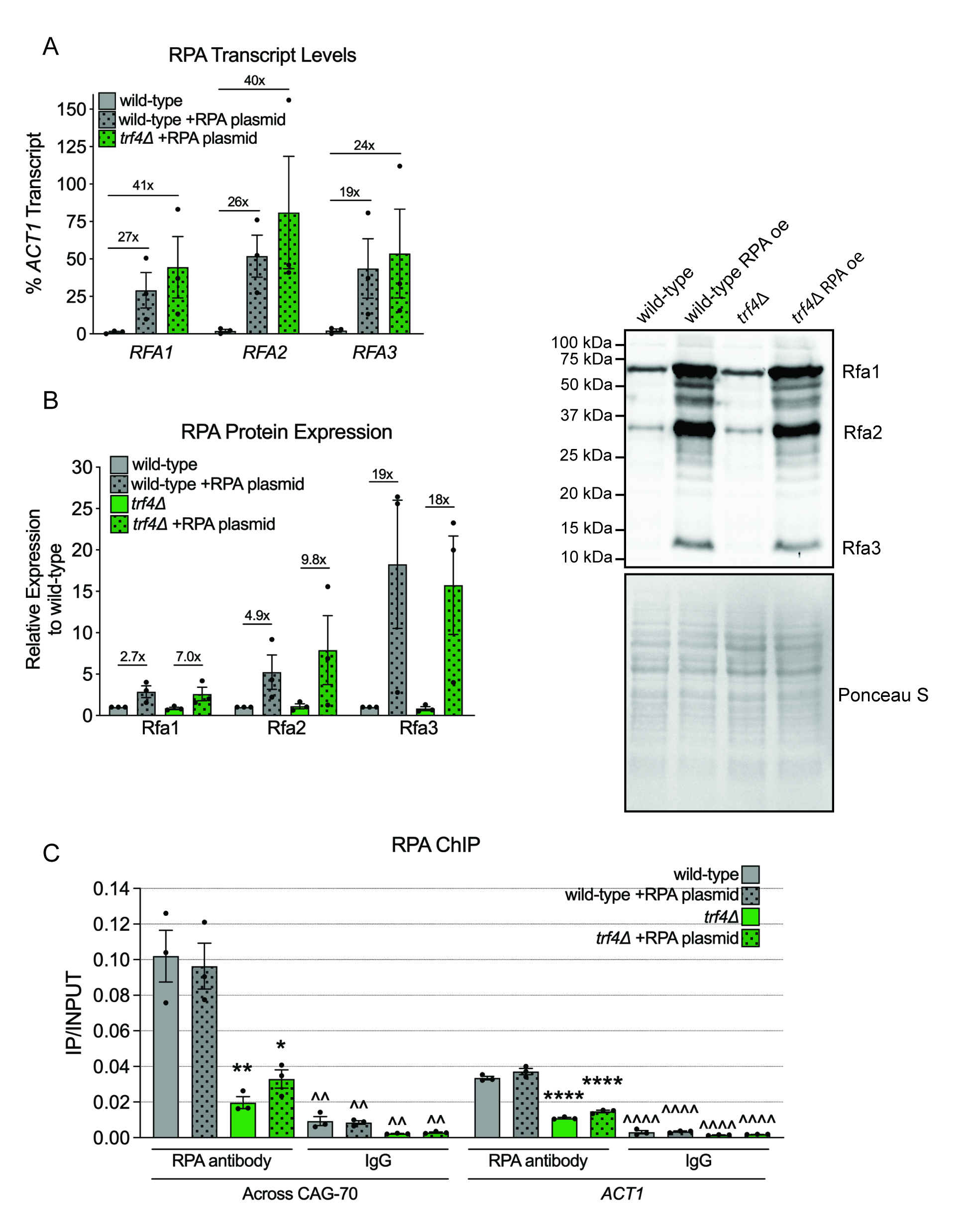

Supplement: S3 Fig — (A) RPA overexpression was achieved by expression of RFA1, RFA2, and RFA3 from 2μ multicopy plasmid (strains transformed with RFA1/RFA2/RFA3 2μ multicopy plasmid compared to no vector). mRNA was reversed transcribed into cDNA by RT-PCR and qPCR was used to quantify cDNA at RFA1, RFA2, RFA3 and ACT1 gene loci. RFA1/RFA2/RFA3 were normalized to the ACT1 qPCR signal in the indicated strains (Table N in S1 Tables). (B) Rfa1, Rfa2, and Rfa3 protein levels quantified in wild-type and trf4Δ strains with and without RPA overexpression (strains transformed with RFA1/RFA2/RFA3 2μ multicopy plasmid compared to no vector). Each data point represents the quantification of Rfa1/Rfa2/Rfa3 protein levels relative to wild type (Table O in S1 Tables). Three separate biological replicates were analyzed for protein quantity. A representative western blot either hybridized to the RPA antibody (detecting Rfa1, Rfa2, and Rfa3) or stained for total protein levels with Ponceau S are shown below the graph. See S1 Raw images for all raw western blot images used for quantification. Different exposures of blots were used to quantify Rfa1, Rfa2, and Rfa3 protein levels (shown is 1 example blot with the exposure used to quantify Rfa3). (C) RPA ChIP shown as IP/INPUT in wild-type and trf4Δ strains with and without RPA overexpression. Use of an RPA antibody for immunoprecipitation and IgG negative controls are shown. Each bar represents the mean ± SEM of at least 3 biological replicates; each data point represents an individual biological replicate. *p < 0.05, **p < 0.01, and ****p < 0.0001 compared to wild-type, ^^p < 0.01 and ^^^^p < 0.0001 compared to corresponding strain with RPA antibody used for IP, by t test (Table M in S1 Tables). (TIF) [file pbio.3001940.s003.tif]

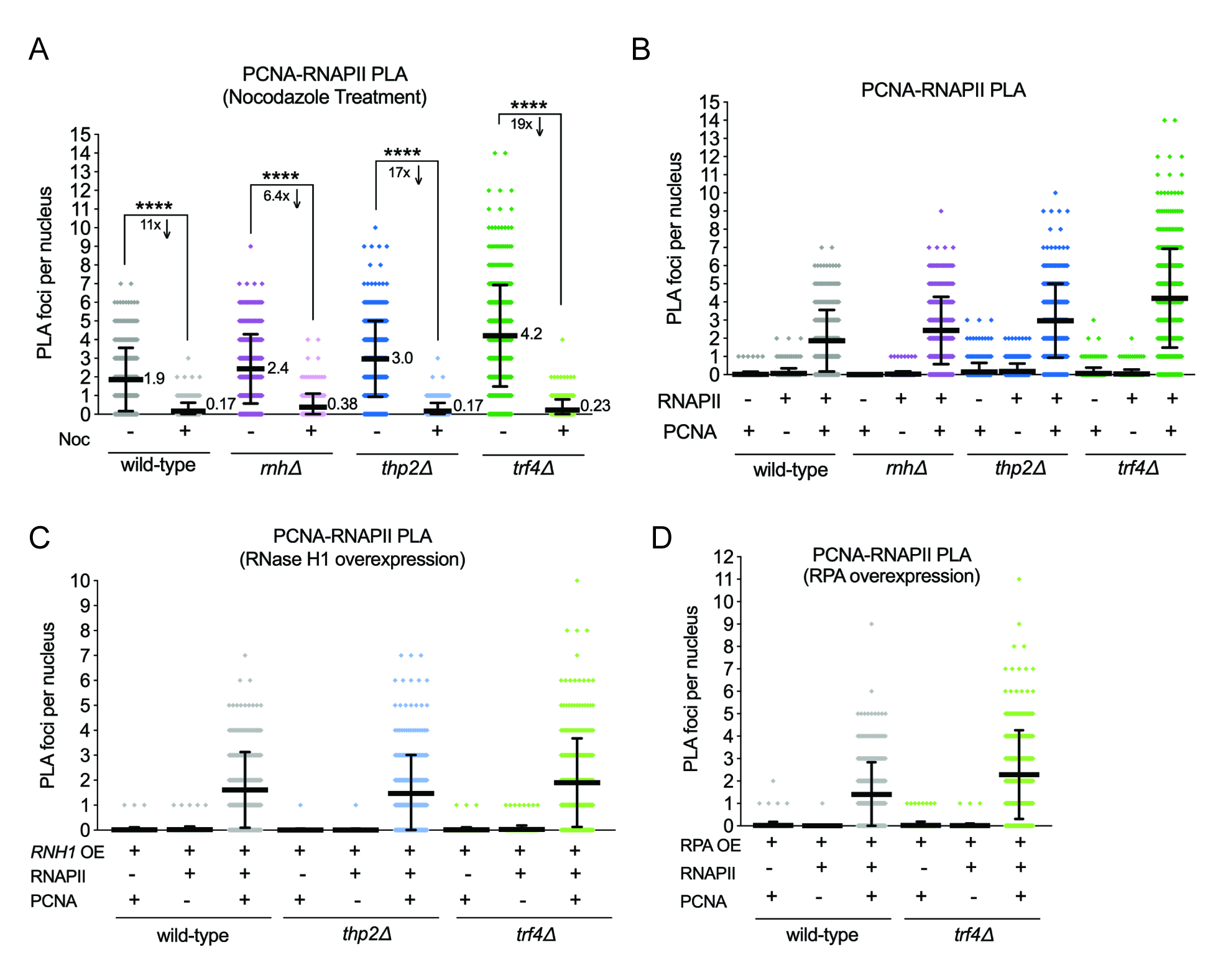

Supplement: S4 Fig — Antibodies to the Ser2 phosphorylated form of RNAPII (RNAPII-pSer2) and one to PCNA were used to assess TRCs genome-wide in the indicated strains. N ≥ 200 (200 for nuclei for nocodazole experiments, 300 for all other experiments) quantified per condition with 100 nuclei screened per replicate. Data points indicate individual PLA foci counts per nucleus (see S2 Tables for raw PLA foci counts). Error bars show mean ± SD. (A) PLA in wild-type, thp2Δ, and trf4Δ strains with nocodazole treatment to reduce TRCs. ****p < 0.0001 comparing no treatment and nocodazole treatment, by Mann–Whitney test. Heavy horizontal bars with adjacent numbers indicate the mean number of foci. (B) PLA in wild-type, rnh1Δrnh201Δ, thp2Δ, and trf4Δ strains double antibody (RNAPII and PCNA) experiments are shown alongside single antibody controls. (C) PLA in wild-type, thp2Δ, and trf4Δ strains either without (RNH1 endogenous promoter) or with RNase H1 overexpression (RNH1 expressed under the PMET25 promoter, induced in the absence of methionine). (D) PLA in wild-type and trf4Δ strains with (strains transformed with RFA1/RFA2/RFA3 2μ multicopy plasmid) and without (strains containing no vector) RPA overexpression. See Table K in S1 Tables for quantification of foci outside nuclei (see S2 Tables for raw PLA foci counts), which averaged 20% and was similar for all strains. See Table L in S1 Tables for p-values of single antibody controls compared to double antibody conditions, by Mann–Whitney test. (TIF) [file pbio.3001940.s004.tif]

**S1 Raw Images. Raw Western Blot Images**

**A.**

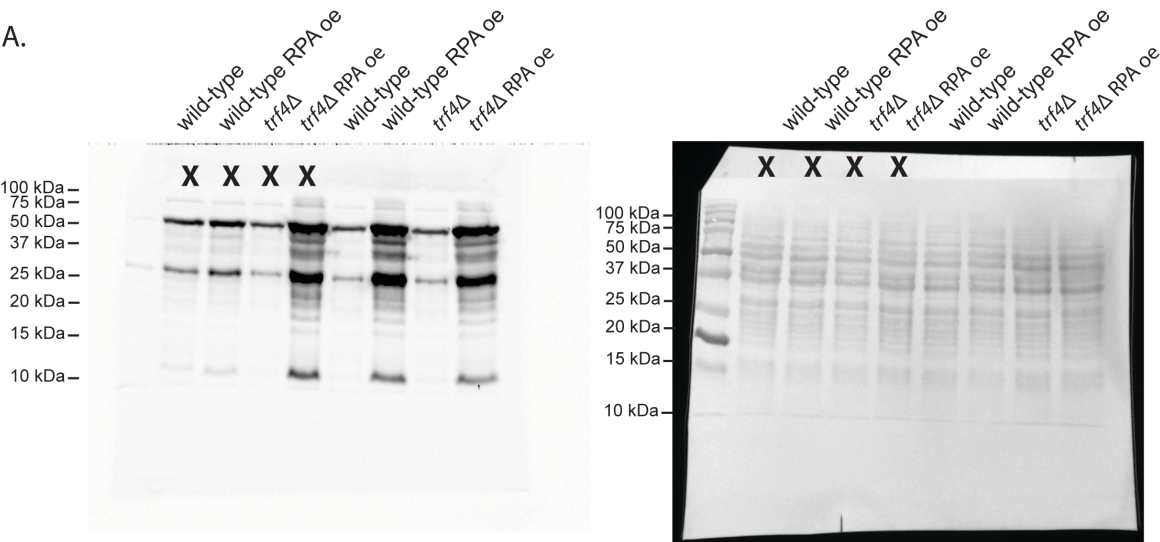

**B.**

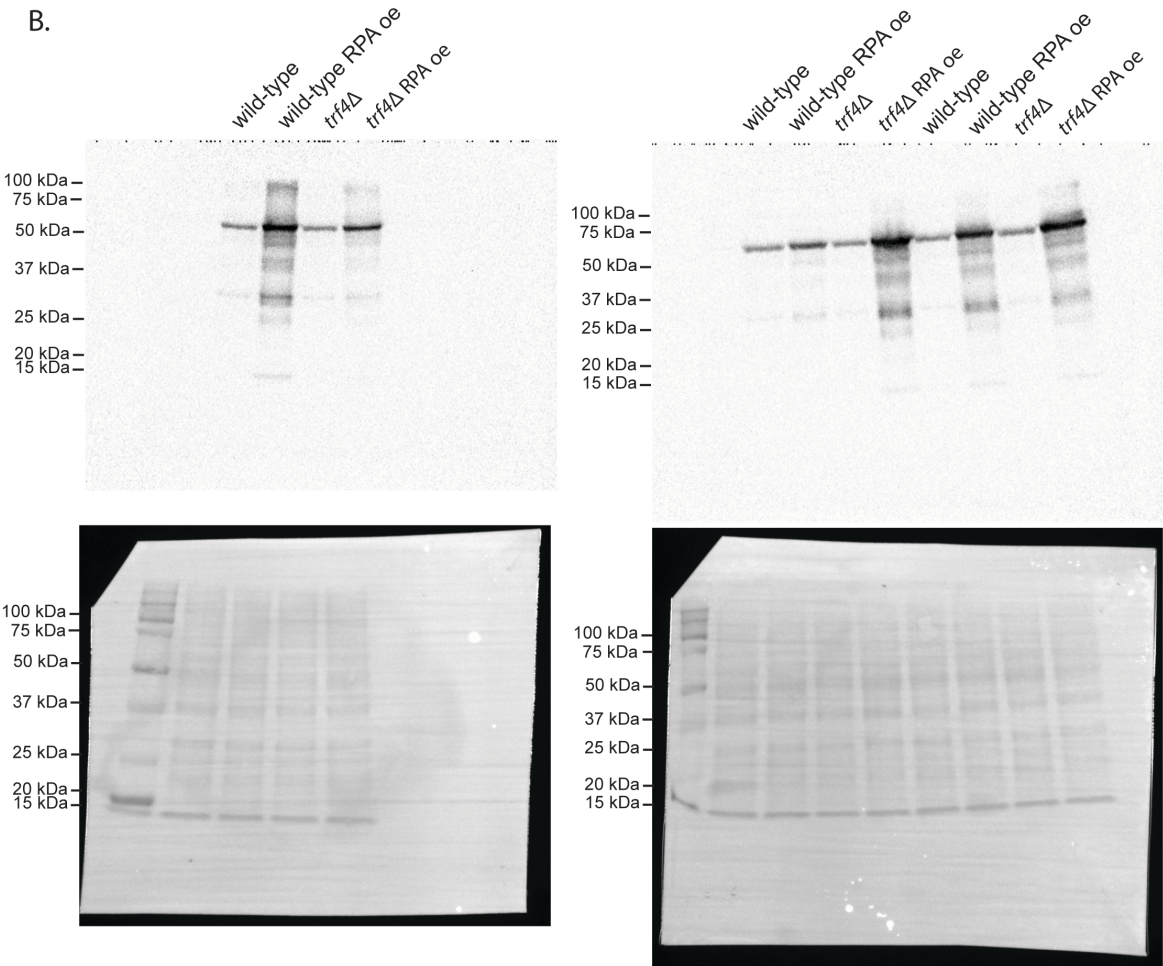

C.

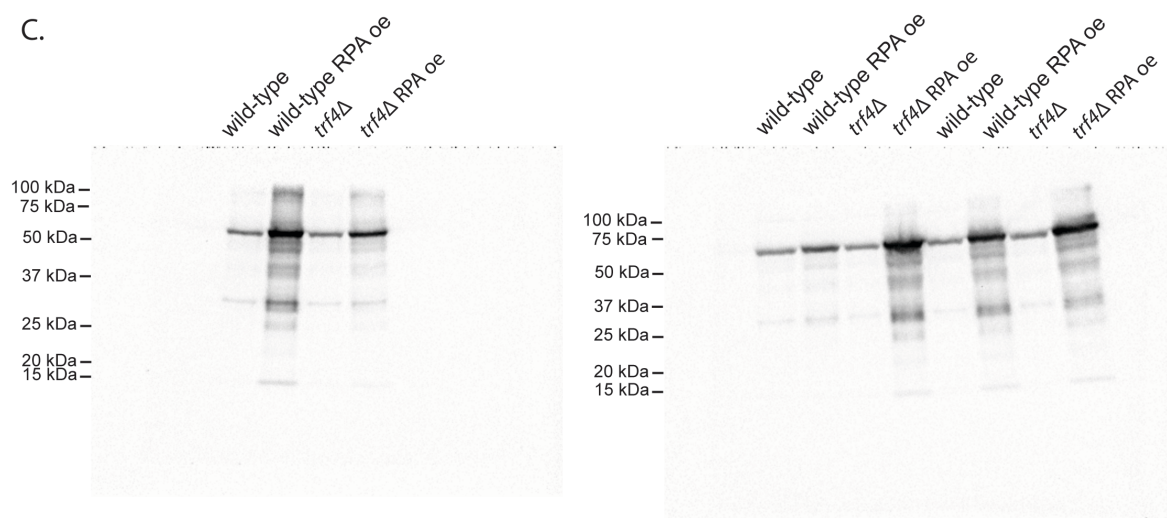

D.

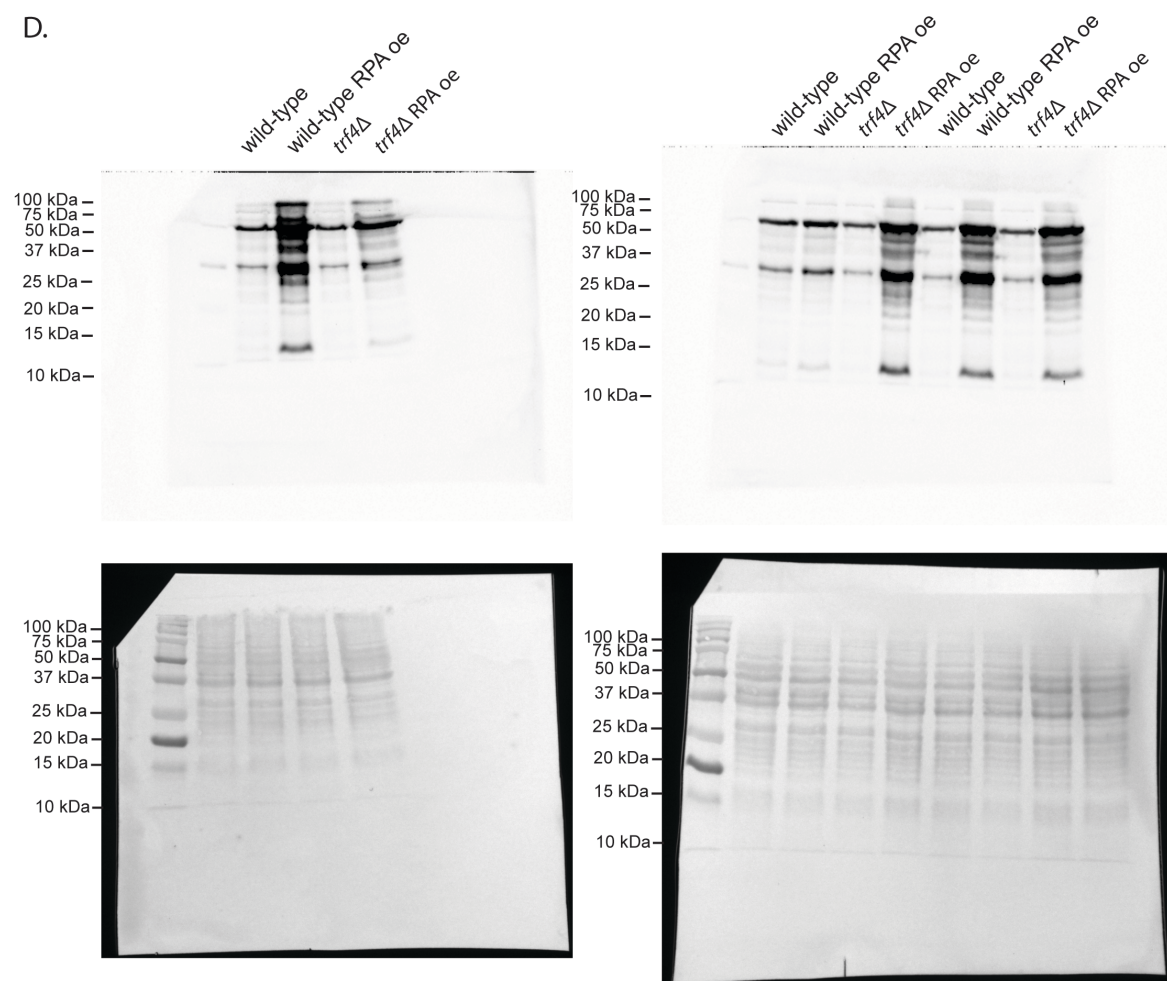

Supplement: S1 Raw images — (A) Raw image of blot shown in S3B Fig. The four lanes on the right (no X) were used for the example blot image shown in S3B Fig. All lanes were used for quantification. Hybridization of RPA antibody is shown on the left and Ponceau S staining is shown on the right. (B) Raw images of blots used for quantification of Rfa1 in S3B Fig and Table O in S1 Tables. Hybridization of RPA antibody is shown above and Ponceau S staining is shown below. (C) Raw images of blots hybridized with RPA antibody used for quantification of Rfa2 in S3B Fig and Table O in S1 Tables. Ponceau S staining shown in panel B was also used for Rfa2 quantification. (D) Raw images of blots used for quantification of Rfa3 in S3B Fig and Table O in S1 Tables. Hybridization of RPA antibody is shown above and Ponceau S staining is shown below. The following method was used to capture the images. For Rfa1/Rfa2/Rfa3 expression: Blots were imaged using the chemi setting on a Bio-Rad ChemiDoc XRS+ Molecular Imager. Appropriate exposure was used for quantification of each of the 3 RPA subunits. For Ponceau S: Blots were imaged using the epi white setting on a Bio-Rad ChemiDoc XRS+ Molecular Imager. (PDF) [file pbio.3001940.s007.pdf]
