# Supplementary material for: The RNA export and RNA decay complexes THO and TRAMP prevent transcription-replication conflicts, DNA breaks, and CAG repeat contractions
Source: PLoS Biol. 2022 Dec 27;20(12):e3001940. doi: 10.1371/journal.pbio.3001940 (PMC9829180; doi:10.1371/journal.pbio.3001940)
Supplement: S1 Tables — Table A. Fragility analysis (rate of FOA-resistance) of CAG-70 repeats on the URA3-YAC. Table B. One-colony fragility analysis (frequency of FOA-resistance) of CAG-70 repeats on the URA3-YAC. Table C. Fragility analysis of no tract (CAG-0) on the URA3-YAC. Table D. Instability analysis of CAG-70 repeats on the URA3-YAC. Table E. Analysis of URA3 presence in FOA-resistant colonies. Table F. Viability of yeast strains on YC-Leu. Table G. DRIP analysis. Table H. CAG-70 transcript expression data. Table I. RNH1 expression data. Table J. RNAPII chromatin immunoprecipitation (ChIP) analysis. Table K. Quantification of foci detected outside of nucleus in PLA experiments. Table L. Proximity-Ligation Assay (PLA) data. Table M. RPA ChIP analysis. Table N. RPA expression data. Table O. RPA protein expression. Table P. Primers used in this study. Table Q. Yeast Strains used in this study. (PDF) [file pbio.3001940.s005.pdf]

S1 Tables. Supporting Information Tables

Table A. Fragility analysis (rate of FOA-resistance) of CAG-70 repeats on the *URA3*-YAC.

**Strains**	**Individual rate of FOA^{R} (x10^{-6})**	**Average rate of FOA^{R} (x10^{-6}) ± S.E.M**	**Fold over wt**	**# of Assays**	**p-value to wt**	**Other p-values a to *rnh1Δrnh201Δ*; b to *thp2Δ*; c to *trf4Δ***
Wild-type	4.2[1]	7.01±1.3	-	5	-	-
	5.2[1]					
	8.5[1]					
	5.97					
	11.2					
rnh1Δrnh201Δ[2]	57.6	48.7±6.9	6.9	3	2.3x10^{-4}	-
	35.1					
	53.4					
thp2Δ	33.4	33.2±3.5	4.7	6	1.1x10^{-4}	0.057[a]
	40.4					
	44.8					
	31.3					
	21.1					
	28.1					
mft1Δ	43.3	47.4±3.9	6.8	4	1.2x10^{-5}	0.87[a]; 0.028[b]
	52.3					
	55.3					
	38.7					
thp2Δrnh1Δrnh201Δ	475	282±71	40.0	4	3.1x10^{-3}	0.039[a]; 2.2X10^{-3}[b]
	187					
	167					
	297					
trf4Δ	39.1	52.8±9.8	7.5	4	1.2x10^{-3}	0.77[a]; 0.059[b]
	62.7					
	75.3					
	33.9					
trf5Δ	12.9	12.4±1.8	1.8	3	0.045	0.018[c]
	9.1					
	15.2					
rrp6Δ	56.5	56.6±0.2	8.1	3	1.1x10^{-7}	0.76[c]
	57					
	56.2					
trf4Δrnh1Δrnh201Δ	340	552±74	74.4	4	9.9x10^{-5}	3.0X10^{-3}[a]; 8.0X10^{-4}[c]
	552					
	699					
	498					
trf4Δrrp6Δ	113	136±28	19.3	4	1.2x10^{-3}	0.030[c]; 0.061 to *rrp6Δ*
	84					

	133<br>214					
rad51Δ	14.9<br>29.9<br>18.9	21.2±4.5	3.0	3	8.3x10^{-3}	-
thp2Δrad51Δ	55.0<br>39.1<br>33.9<br>37.3<br>39.4	40.9±3.6	5.8	5	2.2x10^{-5}	0.16[b]; 0.015 to *rad51Δ*
trf4Δrad51Δ	29.5<br>38.9<br>25.5<br>18.5	28.1±4.3	4.0	4	1.2x10^{-3}	0.06[c]; 0.32 to *rad51Δ*
**Strains with p*MET25-RNH1***						**to +Met condition in the same mutant**
wild-type in +Met	18.2<br>30.9<br>25.7	24.9±3.7	-	4	-	-
wild-type in -Met	16.2<br>32.5<br>30.9	24.9±4.7	-	4	-	0.99
thp2Δ in +Met	36.3<br>26.8<br>34.7	32.6±2.9	-	3	-	-
thp2Δ in -Met	14.0<br>20.8<br>22.7	19.2±2.6	-	3	-	0.027
trf4Δ in +Met	55.0<br>38.1<br>70.6	54.6±9.4	-	3	-	-
trf4Δ in -Met	45.6<br>43.1<br>56.1	48.3±4.0	-	3	-	0.57
**Strains with RPA overexpression vector**						**to no overexpression condition**
Wild-type RPA overexpressed	24.3<br>9.72<br>5.95<br>4.69	11.2±4.5	-	4	-	0.36
trf4Δ RPA overexpressed	34.9<br>23.8	23.2±4.5	-	3	-	0.034

	13.0					
	20.9					

1, indicated wild-type data are from [1]; 2, data from [2]; "-Met" represents yeast synthetic media lacking methionine, which confers a *RNH1* over-expression condition; "+Met" represents yeast synthetic media containing methionine, thus does not induce *RNH1* overexpression (see Fig. S1B).

Table B. One-colony fragility analysis (frequency of FOA-resistance) of CAG-70 repeats on the *URA3*-YAC.

**Strains**	**Individual frequency of FOA^R**	**Average frequency of FOA^R ± S.E.M**	**Fold over wt**	**# of Assays**	**p-value to wt**	**Other p-values: b to *thp2Δ*; c to *trf4Δ***
wild-type	0.0183	0.0106±0.0026	-	4	-	-
	0.0749					
	0.0939					
	0.00758					
thp2Δ	0.0435	0.181±0.084	17	6	0.14	-
	0.117					
	0.594					
	0.149					
	0.092					
	0.0893					
trf4Δ	0.232	0.319±0.031	30	4	5.7×10^{-5}	-
	0.319					
	0.363					
	0.361					
thp2Δtrf4Δ	1900	2300±350	21500	3	5.5×10^{-4}	2.1×10^{-4b}; 5.5×10^{-4c}

Table C. Fragility analysis of no tract (CAG-0) on the *URA3*-YAC.

**Strain**	**Individual rate of FOA^R ($X10^{-6}$)**	**Average rate of FOA^R ($X10^{-6}$) ± S.E.M**	**Fold over wt**	**# of Assays**	**p-value to wt**	**Other p-values c, to *trf4Δ***
Wild-type[1]	2.6	2.2±0.45	-	3	-	-
	2.8					
	1.3					
thp2Δ	5.5	9.1±0.98	4.1	7	2.4×10^{-3}	-
	8.6					
	11.7					
	10.8					
	12.5					
	6.8					

	8.1					
trf4Δ	7.0	10.8±1.8	4.9	3	0.020	-
	14.7					
	10.6					

1, wild-type data are from [1].

Table D. Instability analysis of CAG-70 repeats on the *URA3*-YAC.

		Contractions				Expansions			
**Strain**	**Total rxns**	**#**	**%**	**Fold over wt**	**p-value to wt**	**#**	**%**	**Fold over wt**	**p-value to wt**
wild-type[1]	460	20	4.3	-	-	5	1.1	-	-
thp2Δ	256	29	11.3	2.6	$6.0X10^{-3}$	8	3.1	2.8	0.076
trf4Δ	130	20	15.4	3.6	$1.0X10^{-3}$	4	3.1	2.8	0.11

1, wild-type data are from [1].

Table E. Analysis of *URA3* presence in FOA-resistant colonies.

**Strain**	**Presence of *URA3***		**Total number of FOA^R colonies[1]**	**Percent end loss (no *URA3*)**	**Method used**
	**number**	**percent**			
Wild-type	0	0%	20	100%	PCR
rnh1Δrnh201Δ	0	0%	20	100%	PCR
thp2Δ	0	0%	10	100%	Southern Blot
trf4Δ	0	0%	30	100%	PCR
Wild-type RPA overexpressed	3	7.5%	40	92.5%	PCR
trf4Δ RPA overexpressed	6	15%	40	85%	PCR

1, One FOA^R colony per plate was tested, assuring that each event reported was independent (For 10-colony assays, each culture plated on each FOA-Leu plate is from an individual parent colony). PCR methods and primers used for checking *URA3* locus are listed in Supplementary Table 15. Southern blot method is the same as in [3].

Table F. Viability of yeast strains on YC-Leu.

**Strain**	**% Viability**	**Average % Viability**
wild-type	52.6	76.5
	107	
	70.0	
thp2Δrnh Δ	22.5	27.2
	32.0	
	27.0	
trf4Δrnh Δ	20.0	22.0
	13.0	
	33.0	

thp2Δtrf4Δ	13.0	10.0
	10.0	
	7.00	

Table G. DNA:RNA immunoprecipitation (DRIP) analysis.

		No Treatment			RNase H Treatment		
**Locus**	**Strain**	**IP/INPUT**	**Average IP/INPUT ± SEM**	**p-value compared to wild-type**	**IP/INPUT**	**Average IP/INPUT ± SEM**	**p-value compared to no treatment**
Across CAG-70	wild-type	0.0800	0.0578 ± 0.011	-	0.00374	0.00422 ± 0.00032	0.0087
		0.0442			0.00409		
		0.0492			0.00484		
	thp2Δ	0.0767	0.0604 ± 0.0087	0.862	0.0109	0.00762 ± 0.0017	0.004
		0.0471			0.00508		
		0.0575			0.00685		
	trf4Δ	0.0617	0.0450 ± 0.088	0.417	0.0460	0.0283 ± 0.0088	0.254
		0.0319			0.0185		
		0.0412			0.0205		
	rnh1Δrnh201Δ	0.152	0.112 ± 0.021	0.0845	0.0359	0.0317 ± 0.0026	0.0191
		0.0817			0.0271		
		0.102			0.0320		
CAG-70 Proximal	wild-type	0.0457	0.0430 ± 0.0026	-	0.00511	0.00516 ± 0.000045	0.0048
		0.0404			0.00520		
	thp2Δ	0.0599	0.0568 ± 0.0032	0.0796	0.00732	0.00677 ± 0.00055	0.0041
		0.0536			0.00622		
	trf4Δ	0.0410	0.0386 ± 0.0024	0.338	0.0248	0.0238 ± 0.0010	0.0308
		0.0361			0.0227		
	rnh1Δrnh201Δ	0.0905	0.0904 ± 0.000053	0.0031	0.0319	0.0319 ± 0.000024	<0.0001
		0.0904			0.0319		
MMR1	wild-type	0.0540	0.0445 ± 0.0078	-	0.00199	0.00176 ± 0.00024	0.0055
		0.0506			0.00201		
		0.0290			0.00128		
	thp2Δ	0.0586	0.00479 ± 0.0071	0.764	0.00309	0.00290 ± 0.00042	0.0032
		0.0507			0.00350		
		0.0345			0.00209		

	trf4Δ	0.0229	0.0187 ± 0.0028	0.0360	0.0112	0.00882 ± 0.0013	0.0341
		0.0198			0.00870		
		0.0133			0.00658		
	rnh1Δrnh201Δ	0.0642	0.0548 ± 0.0096	0.454	0.0180	0.0135 ± 0.0030	0.0149
		0.0646			0.0147		
		0.0356			0.00787		
PMA1	wild-type	0.0815	0.0896 ± 0.0072	-	0.136	0.0582 ± 0.028	0.319
		0.0918			0.01418		
		0.0762			0.0209		
		0.109			0.0625		
	thp2Δ	0.0880	0.115 ± 0.0198	0.276	0.0412	0.0157 ± 0.0086	0.0037
		0.127			0.00552		
		0.0791			0.00591		
		0.165			0.0104		
	trf4Δ	0.0812	0.127 ± 0.027	0.236	0.0824	0.0350 ± 0.016	0.0273
		0.154			0.0195		
		0.0815			0.0122		
		0.190			0.0258		
	rnh1Δrnh201Δ	0.221	0.285 ± 0.053	0.0103	0.1327	0.0594 ± 0.025	0.008
		0.441			0.0307		
		0.221			0.0250		
		0.258			0.0492		

All IP/INPUT data shown is the average of two technical replicates.

Table H. CAG-70 transcript expression data.

Locus	Strain	Absolute Quantity	Average ± SEM	p-value to wild-type	Fold over wild-type
CAG-70 (rCUG)	wild-type	386	332 ± 29	-	-
		323			
		288			
	thp2Δ	254	247 ± 29	0.106	0.7
		195			
		294			
	trf4Δ	407	420 ± 16	0.0577	2.1
		400			
		452			
CAG-70 (rCAG)	wild-type	68.8	61.8 ± 5.8	-	-
		50.3			
		66.3			
	thp2Δ	44.8	41.8 ± 11	0.172	0.7

		22.2			
		58.4			
	trf4Δ	116	127 ± 15	0.0145	1.7
		109			
		156			

All expression data shown is the average of two technical replicates.

Table I. *RNH1* expression data.

**Strain**	**% *ACT1* (by Absolute Quantity)**	**% *ACT1* Average**	**Fold over endogenous *RNH1***	**Fold over no *RNH1* induction (+Met condition)**
wild-type	2.16	1.90	-	0.205
	1.76			
	1.77			
wild-type P_{MET25}-*RNH1* (+Met)	9.7	9.29	4.89	-
	8.88			
wild-type P_{MET25}-*RNH1* (-Met)	28.6	30.1	15.8	3.24
	31.5			
thp2Δ	1.6	1.56	-	0.231
	1.72			
	1.37			
thp2Δ P_{MET25}-*RNH1* (+Met)	9.75	6.74	4.32	-
	3.72			
thp2Δ P_{MET25}-*RNH1* (-Met)	31.7	32.8	21.0	4.87
	33.9			
trf4Δ	1.55	1.71	-	0.188
	1.8			
	1.79			
trf4Δ P_{MET25}-*RNH1* (+Met)	12.4	9.1	5.32	-
	5.75			
trf4Δ P_{MET25}-*RNH1* (-Met)	30.6	31	18.1	3.41
	31.3			

All % ACT1 data shown is the average of two technical replicates.

Table J. RNAPII chromatin immunoprecipitation (ChIP) analysis.

**Locus**	**Strain**	**IP/INPUT**	**Average IP/INPUT ± SEM**	**p-value compared to wild-type; compared to no RNH1 oe**
CAG-70	wild-type	0.0217	0.0247 ± 0.0072	-
		0.0141		

		0.0384		
	thp2Δ	0.143	0.108 ± 0.027	0.0427 ; -
		0.0538		
		0.1270		
	trf4Δ	0.0438	0.0572 ± 0.0086	0.0442 ; -
		0.0733		
		0.5460		
	rnh1Δrnh201Δ	0.0386	0.0383 ± 0.0016	0.0837 ; -
		0.0337		
		0.0408		
		0.0401		
	wild-type pMET25-RNH1	0.0291	0.0258 ± 0.0057	0.910
		0.0336		
		0.0148		
	thp2Δ pMET25-RNH1	0.0490	0.0428 ± 0.0034	0.0854 ; 0.0783
		0.0371		
		0.424		
	trf4Δ pMET25-RNH1	0.0435	0.0401 ± 0.0029	0.119 ; 0.132
		0.0343		
		0.424		
	PGAL1-CAG-100 (glucose)	0.0266	0.0376 ± 0.0087	0.372 ; -
		0.0314		
		0.0548		
	PGAL1-CAG-100 (galactose)	0.261	0.226 ± 0.025	0.0016 ; 0.0021 (compared to glucose)
		0.241		
		0.177		
ACT1	wild-type	0.00989	0.00976 ± 0.0027	-
		0.00499		
		0.0144		
	thp2Δ	0.0453	0.0896 ± 0.045	0.162 ; -
		0.0386		
		0.1770		
	trf4Δ	0.0895	0.0774 ± 0.011	0.0035 ; -
		0.0863		
		0.0563		
	rnh1Δrnh201Δ	0.0216	0.0268 ± 0.0027	0.0078 ; -
		0.0225		
		0.0323		
		0.0306		

	wild-type pMET25-RNH1	0.0125	0.0248 ± 0.011	0.259
		0.0150		
		0.0470		
	thp2Δ pMET25-RNH1	0.0234	0.0278 ± 0.0026	0.0086 ; 0.260
		0.0323		
		0.0276		
	trf4Δ pMET25-RNH1	0.0465	0.0362 ± 0.0097	0.0586 ; 0.0458
		0.0168		
		0.0454		
MMR1	wild-type	0.00740	0.00598 ± 0.0019	-
		0.00221		
		0.0083		
	thp2Δ	0.0286	0.0319 ± 0.012	0.104 ; -
		0.0126		
		0.0544		
	trf4Δ	0.0286	0.0301 ± 0.0013	0.0005 ; -
		0.0326		
		0.0290		
	rnh1Δrnh201Δ	0.00422	0.0101 ± 0.00042	0.0571 ; -
		0.00186		
		0.00318		
		0.00229		
	wild-type pMET25-RNH1	0.0105	0.0125 ± 0.0018	0.0654
		0.0110		
		0.0160		
	thp2Δ pMET25-RNH1	0.0108	0.0140 ± 0.0018	0.0385 ; 0.221
		0.0172		
		0.0141		
	trf4Δ pMET25-RNH1	0.0297	0.0212 ± 0.0060	0.0720 ; 0.218
		0.00970		
		0.0241		
OLI1	wild-type	0.00255	0.00277 ± 0.00057	-
		0.00191		
		0.00384		
	thp2Δ	0.00242	0.00106 ± 0.00071	0.132 ; -
		0.0000560		
		0.000698		
	trf4Δ	0.00164	0.000890 ± 0.00038	0.0509 ; -
		0.000540		

		0.000490		
	rnh1Δrnh201Δ	0.0386	0.00289 ± 0.00052	0.883 ; -
		0.0337		
		0.0408		
		0.0401		
	wild-type pMET25-RNH1	0.0012	0.00175 ± 0.00034	0.199
		0.0017		
		0.0024		
	thp2Δ pMET25-RNH1	0.000355	0.000600 ± 0.00020	0.0227 ; 0.566
		0.000456		
		0.000991		
	trf4Δ pMET25-RNH1	0.00483	0.00221 ± 0.0013	0.720 ; 0.390
		0.000578		
		0.00123		

All IP/INPUT data shown is the average of two technical replicates.

Table K. Quantification of foci detected outside of nucleus in PLA experiments.

Strains	Average # foci in nucleus	Average # foci outside nucleus	Total # foci outside of nucleus	Total # of foci inside nucleus	Ratio of foci outside vs. inside nucleus
wild-type	1.9	0.55	164	559	0.29
rnh1Δrnh201Δ	2.4	0.46	139	730	0.19
thp2Δ	3	0.61	184	890	0.21
trf4Δ	4.2	1.2	465	1682	0.28
wild-type pMET25-RNH1	1.61	0.24	72	484	0.15
thp2Δ pMET25-RNH1	1.47	0.17	50	442	0.11
trf4Δ pMET25-RNH1	1.90	0.34	102	571	0.18
wild-type RPA overexpressed	1.40	0.26	79	420	0.19
trf4Δ RPA overexpressed	2.29	0.53	158	686	0.23
wild-type +Nocodazole	0.165	0.070	14	33	0.42
rnh1Δrnh201Δ +Nocodazole	0.380	0.17	33	76	0.43
thp2Δ +Nocodazole	0.170	0.065	13	34	0.38
trf4Δ +Nocodazole	0.225	0.070	14	45	0.31

Foci outside of the nucleus were not counted in the total # of foci presented in Table S9. We quantified these foci to ensure the percentage of foci outside of the nucleus did not vary significantly between strains.

Table L. Proximity-Ligation Assay (PLA) data.

Strain	Antibody	Total # of cells	Total # of foci	Average foci per nucleus	Median foci per nucleus	Standard deviation	p-value compared to wild-type (or no treatment)	p-value compared to no RNH1 or RPA over-expression	p-value compared to double antibody condition
wild-type	Both	300	559	1.86	2	1.7	-	-	-
	PCNA only	300	6	0.0200	0	0.14	-	-	<0.0001
	RNAPII only	300	20	0.0667	0	0.29	-	-	<0.0001
thp2Δ	Both	300	890	2.97	3	2.0	<0.0001	-	-
	PCNA only	300	44	0.147	0	0.50	-	-	<0.0001
	RNAPII only	300	50	0.167	0	0.44	-	-	<0.0001
trf4Δ	Both	400	1682	4.21	4	2.7	<0.0001	-	-
	PCNA only	300	21	0.0700	0	0.31	-	-	<0.0001
	RNAPII only	300	14	0.0467	0	0.23	-	-	<0.0001
rnh1Δrnh201Δ	Both	300	730	2.43	2	1.9	0.0001	-	-
	PCNA only	300	0	0	0	0	-	-	<0.0001
	RNAPII only	300	8	0.0267	0	0.16	-	-	<0.0001
wild-type pMET25-RNH1	Both	300	484	1.61	1	1.5	0.11	0.11	-
	PCNA only	300	3	0.0100	0	0.10	-	-	<0.0001
	RNAPII only	300	5	0.0167	0	0.13	-	-	<0.0001
thp2Δ pMET25-RNH1	Both	300	442	1.47	1	1.5	0.0038	<0.0001	-
	PCNA only	300	1	0.00333	0	0.058	-	-	<0.0001
	RNAPII only	300	1	0.00333	0	0.058	-	-	<0.0001
trf4Δ pMET25-RNH1	Both	300	571	1.90	2	1.8	0.89	<0.0001	-
	PCNA only	300	3	0.0100	0	0.10	-	-	<0.0001

	RNAPII only	300	8	0.0267	0	0.16	-	-	<0.0001
wild-type RPA overexpressed	Both	300	420	1.40	1	1.4	0.0011	0.0011	-
	PCNA only	300	6	0.0200	0	0.16	-	-	<0.0001
	RNAPII only	300	1	0.00333	0	0.058	-	-	<0.0001
trf4Δ RPA overexpressed	Both	300	686	2.29	2	2.0	0.014	<0.0001	-
	PCNA only	300	8	0.0267	0	0.16	-	-	<0.0001
	RNAPII only	300	3	0.0100	0	0.10	-	-	<0.0001
wild-type +Nocodazole	Both	200	33	0.165	0	0.46	<0.0001	-	-
rnh1Δrnh201Δ +Nocodazole	Both	200	76	0.380	0	0.73	<0.0001	-	-
thp2Δ +Nocodazole	Both	200	34	0.170	0	0.44	<0.0001	-	-
trf4Δ +Nocodazole	Both	200	45	0.225	0	0.56	<0.0001	-	-

Table M. RPA ChIP analysis.

		RPA Antibody		
		**IP/INPUT**	**Average IP/INPUT ± SEM**	**p-value compared to wild-type; no RPA oe**
CAG-70	wild-type	0.104	0.102 ± 0.015	-
		0.126		
		0.0758		
	trf4Δ	0.0163	0.0197 ± 0.0033	0.0053
		0.0263		
		0.0166		
	wild-type +RPA overexpression	0.0905	0.0963 ± 0.013	0.0786
		0.121		
		0.0775		
	trf4Δ + RPA overexpression	0.0233	0.0330 ± 0.0051	0.0110; 0.0946
		0.0407		
		0.0348		
ACT1	wild-type	0.0322	0.0335 ± 0.0010	-
		0.0328		
		0.0355		
	trf4Δ	0.0117	0.0109 ± 0.00041	<0.0001

		0.0106		
		0.0104		
	wild-type +RPA overexpression	0.0341	0.0371 ± 0.0017	0.144
		0.0372		
		0.0401		
	trf4Δ + RPA overexpression	0.0155	0.0147 ± 0.00065	<0.0001; 0.0076
		0.0153		
		0.0135		
		**IgG**		
		**IP/INPUT**	**Average IP/INPUT ± SEM**	**p-value compared to wild-type; no RPA oe**
CAG-70	wild-type	0.0143	0.00932 ± 0.0025	-
		0.00748		
		0.00613		
	trf4Δ	0.00213	0.00223 ± 0.00016	0.0497
		0.00254		
		0.00202		
	wild-type +RPA overexpression	0.00896	0.00850 ± 0.0010	0.781
		0.0100		
		0.00658		
	trf4Δ + RPA overexpression	0.00234	0.00287 ± 0.00035	0.0660; 0.166
		0.00276		
		0.00352		
ACT1	wild-type	0.00489	0.00305 ± 0.00095	-
		0.00252		
		0.00174		
	trf4Δ	0.00163	0.00149 ± 0.00011	0.177
		0.00128		
		0.00156		
	wild-type +RPA overexpression	0.00371	0.00340 ± 0.00034	0.748
		0.00378		
		0.00271		
	trf4Δ + RPA overexpression	0.00176	0.00189 ± 0.000067	0.288; 0.0341
		0.00197		
		0.00195		

All IP/INPUT data shown is the average of two technical replicates.

Table N. RPA expression data.

Strain	% *ACT1* (by Absolute Quantity)	% *ACT1* Average	Fold Change over wild-type (by Absolute Quantity)	Fold change over wild-type Average
RFA1				
wild-type +RPA plasmid	26.8	29.1	20.0	26.6
	9.85		193	
	50.6		26.7	
trf4Δ +RPA plasmid	37.1	44.5	27.6	40.7
	13.3		261	
	83.1		43.9	
wild-type (no plasmid)	1.34	1.09	1	-
	0.0511		1	
	1.89		1	
RFA2				
wild-type +RPA plasmid	52.4	52.0	22.7	25.8
	27.4		155	
	76.0		21.3	
trf4Δ +RPA plasmid	46.0	81.0	19.9	40.1
	40.9		230	
	156.0		43.7	
wild-type (no plasmid)	2.31	2.02	1	-
	0.177		1	
	3.57		1	
RFA3				
wild-type +RPA plasmid	37.2	43.7	12.5	19.4
	13.1		90.6	
	80.7		22.2	
trf4Δ +RPA plasmid	33.4	53.7	11.2	23.8
	15.5		107	
	112		30.9	
wild-type (no plasmid)	2.98	2.25	1	-
	0.145		1	
	3.63		1	

All % ACT1 data shown is the average of two technical replicates.

Table O. RPA protein expression.

	Rfa1	Rfa2	Rfa3	Ponceau S (for Rfa1/Rfa2)	Ponceau S (for Rfa3)	
**wild-type**	1.00	1.00	1.00	1.00	1.00	
	1.00	1.00	1.00	1.00	1.00	
	1.00	1.00	1.00	1.00	1.00	
**wild-type RPA oe**	3.01	4.31	25.59	1.22	0.95	
	1.57	2.20	2.78	1.04	0.74	
	4.02	9.20	26.41	1.01	0.96	
trf4Δ	0.780	0.640	0.66	1.72	0.82	
	0.650	1.03	0.52	2.28	0.75	
	1.13	1.67	1.31	0.73	1.26	
***trf4Δ* RPA oe**	1.57	1.25	3.98	1.41	0.93	
	2.01	15.56	19.98	2.36	1.16	
	4.19	6.89	23.26	0.30	0.99	
	**Rfa1 Norm to Ponceau S**	**Rfa2 Norm to Ponceau S**	**Rfa3 Norm to Ponceau S**	**Average Rfa1 Norm to Ponceau S**	**Average Rfa2 Norm to Ponceau S**	**Average Rfa3 Norm to Ponceau S**
**wild-type**	1.00	1.00	1.00	1.00	1.00	1.00
	1.00	1.00	1.00			
	1.00	1.00	1.00			
**wild-type RPA oe**	2.47	3.53	26.94	2.65	4.92	19.4
	1.46	2.12	3.76			
	3.98	9.11	27.51			
trf4Δ	0.45	0.37	0.80	0.76	1.04	0.85
	0.29	0.45	0.69			
	1.55	2.29	1.04			
***trf4Δ* RPA oe**	1.11	0.89	4.28	5.31	10.15	15.0
	0.85	6.59	17.22			
	13.97	22.97	23.49			

Table P. Primers used in this study.

Locus	Primer name	Oligonucleotide sequence
Cross CAG (instability)	NewCAGfor	CCTCAGCCTGGCCGAAAGAAAGAAA
	NewCAGrev	CAGTCACGACGTTGTAAAACGACGG
Cross CAG (confirming tract length for fragility assays)	CAG-2Step-F	GCGTGGAGGATGGAACACGGACGG
	CAG-2Step-R	GAAAGGGGGATGTGCTGCAAGGCG
Cross CAG	T7-20B	GAATTCGAGCTCCACCGCGG

(qPCR)	CTG rev2	CCCAGGCCTCCAGTTTGC
CAG proximal (upstream) (qPCR)	CAGproxFW2	TCGCCCTATAGTGAGTCGTATTA
	CAGproxRev2	TGTGCTGCAAGGCGATTA
CAG proximal (downstream) (qPCR)	pGEM CAG For	GCTTGGCGTAATCATGGTC
	pGEM CAG Rev	GCAGTGAGCGCAACGCAAT
CAG locus specific primers (cDNA synthesis for rCUG/rCAG)	CAGproxRev2	TGTGCTGCAAGGCGATTA
	pGEM CAG Rev	GCAGTGAGCGCAACGCAAT
URA3 internal (check presence)	URA3 for2	TGCTGCTACTCATCCTAG
	URA3 rev	TCCCAGCCTGCTTTTCTGTA
URA3 internal (qPCR)	URA3internalFor	AATTGCAGTACTCTGCGGGT
	URA3internalRev	AGGCCTCTAGGTTCCTTTGT
RFA1 internal (qPCR)	RFA1_qPCR_F	TCGTGTGACGGATTTTGGTGG
	RFA1_seq_R2	GATGAAGTTTGCGTTGCGGC
RFA2 internal (qPCR)	RFA2_qPCR_F	TCACGTTTGTTTTGTAGGTGTGG
	RFA2_seq_R	CATCGTTACCGGCAGCCAAG
RFA3 internal (qPCR)	RFA3_seq_F	CGAAACACCAAGAGTTGACCCC
	RFA3_qPCR_R	CGTAATCATTTCAACCTCGCTGCC
PMA1 internal (qPCR)	PMA1 3' F	TACTGTCGTCCGTGTCTGGATCT
	PMA1 3' R	CCTTCATTGGCTTACCGTTCA
MMR1 internal (qPCR)	MMR1 internal for	GCCCTAAGACTAGACTGGCAC
	MMR1 internal rev	GCAGAAGTTGGCTCCTCTTC
ACT1 internal (qPCR)	ACT1for3	TCCAGATGGTCAAGTCATCA
	ACT1rev3	TCGGCAATACCTGGGAACAT
RNH1 internal (qPCR)	RNH1-upreg-verif-Reverse	GCTTGTAATCATGCGCACTCATAC
	RNH1 internal forward 1	GCGAGTTCATCGAAGGAATCGGC

Table Q. Yeast Strains used in this study.

Strain Number	Strain Background	Genotype	Reference
CFY765	BY4705	MATα, *ade2Δ::hisG*, *his3Δ200*; *leu2Δ0, lys2Δ0, met15Δ0, trp1Δ63, ura3Δ0, can*^{R}; YAC: *ade3-2p, LEU2,* CAG-0, *URA3*.	[1, 4]
CFY766	BY4705	MATα, *ade2Δ::hisG*, *his3Δ200*; *leu2Δ0, lys2Δ0, met15Δ0, trp1Δ63, ura3Δ0, can*^{R}; YAC: *ade3-2p, LEU2,* CAG-70,*URA3*.	[1, 4]
CFY3418, 3419	BY4705	CFY766, *rnh1Δ::His3MX6*; *rnh201Δ::TRP1*	[2]
CFY2393, 2394	BY4705	CFY765, *thp2Δ::KanMX6*	This Study
CFY2395, 2396	BY4705	CFY766, *thp2Δ::KanMX6*	This Study
CFY4094, 4095	BY4705	CFY766, *mft1Δ::KanMX6*	This Study
CFY3416, 3417	BY4705	CFY3418, *thp2Δ::KanMX6*	This Study

CFY1976, 1977	BY4705	CFY765, *trf4Δ::His3MX6*	This Study
CFY1863	BY4705	CFY766, *trf4Δ::KanMX6*	This Study
CFY4113,4 127	BY4705	CFY3418 *trf4Δ::KanMX6*	This Study
CFY4111, 4112	BY4705	CFY2395 *trf4Δ::His3MX6*	This Study
CFY2044, 2045	BY4705	CFY766 *trf5Δ::His3MX6*	This Study
CFY4224, 4225	BY4705	CFY766 *rrp6Δ::TRP1*	This Study
CFY4324, 4325	BY4705	CFY1977 *rrp6Δ::TRP1*	This Study
CFY3466	BY4705	MATα, *ade2Δ::hisG*, *his3Δ200*; l*eu2Δ0, lys2Δ0, trp1Δ63, ura3Δ0, can*^{R}; YAC: *ade3-2p, LEU2,* CAG-70,*URA3*; *pMET25-RNH1*; *thp2Δ::KanMX6*	This Study
CFY4343, 4344	BY4705	MATα, *ade2Δ::hisG*, *his3Δ200*; *leu2Δ0, lys2Δ0, trp1Δ63, ura3Δ0, can*^{R}; YAC: *ade3-2p, LEU2,* CAG-70,*URA3*; *pMET25-RNH1*; *trf4Δ:: His3MX6*	This Study
CFY4334	BY4705	MATα, *ade2Δ::hisG*, *his3Δ200*; *leu2Δ0, lys2Δ0, trp1Δ63, ura3Δ0, can*^{R}; YAC: *ade3-2p, LEU2,* CAG-70,*URA3*; *pMET25-RNH1*	This Study
CFY5659, 5660, 5661	BY4705	CFY766, transformed with pUC57 RPA OE *HIS3* plasmid containing *RFA1, RFA2, RFA3* genes (pUC57 made by S. Khristich, plasmid stock CFP772)	This Study, [5]
CFY5662, 5663, 5664	BY4705	CFY1863, transformed with pUC57 RPA OE *HIS3* plasmid containing *RFA1, RFA2, RFA3* genes	This Study, [5]

References

1. Kerrest A, Anand RP, Sundararajan R, Bermejo R, Liberi G, Dujon B, et al. SRS2 and SGS1 prevent chromosomal breaks and stabilize triplet repeats by restraining recombination. Nat Struct Mol Biol. 2009;16(2):159-67. doi: 10.1038/nsmb.1544. PubMed PMID: 19136956; PubMed Central PMCID: PMCPMC4454460.
2. Su XA, Freudenreich CH. Cytosine deamination and base excision repair cause R-loop-induced CAG repeat fragility and instability in Saccharomyces cerevisiae. Proc Natl Acad Sci U S A. 2017;114(40):E8392-E401. doi: 10.1073/pnas.1711283114. PubMed PMID: 28923949; PubMed Central PMCID: PMCPMC5635916.
3. Callahan JL, Andrews KJ, Zakian VA, Freudenreich CH. Mutations in yeast replication proteins that increase CAG/CTG expansions also increase repeat fragility. Mol Cell Biol. 2003;23(21):7849-60. PubMed PMID: 14560028; PubMed Central PMCID: PMCPMC207578.
4. Sundararajan R, Gellon L, Zunder RM, Freudenreich CH. Double-strand break repair pathways protect against CAG/CTG repeat expansions, contractions and repeat-mediated chromosomal fragility in Saccharomyces cerevisiae. Genetics. 2010;184(1):65-77. doi: 10.1534/genetics.109.111039. PubMed PMID: 19901069; PubMed Central PMCID: PMCPMC2815931.

5. Khristich AN, Armenia JF, Matera RM, Kolchinski AA, Mirkin SM. Large-scale contractions of Friedreich's ataxia GAA repeats in yeast occur during DNA replication due to their triplex-forming ability. Proc Natl Acad Sci U S A. 2020;117(3):1628-37. Epub 2020/01/09. doi: 10.1073/pnas.1913416117. PubMed PMID: 31911468; PubMed Central PMCID: PMCPMC6983365.
