# Supplementary material for: The RNA export and RNA decay complexes THO and TRAMP prevent transcription-replication conflicts, DNA breaks, and CAG repeat contractions
Source: PLoS Biol. 2022 Dec 27;20(12):e3001940. doi: 10.1371/journal.pbio.3001940 (PMC9829180; doi:10.1371/journal.pbio.3001940)
Supplement: Attachment — Submitted filename: Response to Reviewers_Brown,Su...Freudenreich.docx [file pbio.3001940.s008.docx]

Response to Reviewers

Title: THO and TRAMP complexes prevent transcription-replication conflicts, DNA breaks, and CAG repeat contractions

Authors: Rebecca E. Brown, Xiaofeng A. Su, Stacey Fair, Katherine Wu, Lauren Verra, Robyn Jong, Kristin Andrykovich, Catherine H. Freudenreich

We would like to thank the editor and reviewers for their thoughtful evaluation of our manuscript. All major text changes and those requested in the below reviews are in red text in the revised manuscript. The additional experiments included in the revised manuscript are listed below and also addressed within the responses to the reviewer comments.

**Experimentally We Have Added:**

- Repeated the DRIP with requested changes (no crosslinking, no sonication, including RNase H treatment as a control, and including a locus as a positive control for R-loop formation in THO mutants). *Result:* *same results as original protocol and all signals were reduced significantly by RNaseH treatment, indicating hybrid detection. We noted that the reduction was less in trf4 strains though, which may indicate some dsRNA detection by S9.6 in that strain, consistent with our model*. (New Fig. 3B, S2A, S2B)
- Quantified rCUG and rCAG transcript levels in wild-type, *thp2Δ*, and *trf4Δ* strains. *Result:* *trf4 strains show a slight increase, otherwise no effects*. (New Fig. 3C)
- Re-did the RNAPII ChIP with/without RNase H1 overexpression in wild-type, *thp2Δ*, and *trf4Δ* strains, showing IP/Input for the CAG locus and 3 other loci, including a positive and negative control. *Result:* *RNaseH overexpression* *releases RNAPII in thp2Δ mutant but no significant effect in trf4Δ mutant*. (New Fig. 3E, S2C)
  - Note: the new RNAPII ChIP data strengthens our conclusions about RNAPII stalling in the *thp2Δ* mutant (now see 4.4-fold over wild-type compared with old data of 2.9-fold). We got a similar fold-over wild-type for the *trf4Δ* mutant (2.3-fold for new data and 2.7-fold for old data) but have now added the caveat that this could also be due to increased transcription in that mutant, based on the new data in Fig. 3C (though we think the increase in transcript levels is more likely due to a defect in elimination of cryptic transcripts in the *trf4Δ* mutant)
  - A difference with the previous data is that we now also detect an increase in RNAPII at the *ACT1* and (newly tested) *MMR1* loci in both mutants. We feel our new RNAPII ChIP data is of better quality so have replaced the old data and adjusted conclusions accordingly.
- Repeated the RPA ChIP with/without RPA overexpression in wild-type and *trf4Δ* strains, showing IP/Input for both the CAG locus and the ACT1 locus and the IgG control. We feel our new RPA ChIP data is better quality and have replaced the old data as well. *Result: As before, observed a significant (now 5.2-fold) decrease in RPA at the CAG locus. There is also a 3.1x decrease at the ACT1 locus, consistent with the genome-wide effects observed for increased TRCs by PLA.* (Fig. 5A & S3C). Revised to text to emphasize that decreased RPA binding is likely a genome-wide effect.
- Performed a western blot to detect Rfa1, Rfa2, Rfa3 protein levels to confirm that RPA overexpression increases both RPA RNA and protein levels (*it does, 3-20-fold depending on subunit*). (Fig. S3B)
- Treated cells with nocodazole to examine if PLA foci/TRCs are reduced in cells that are not in S phase. *Result:* *PLA foci are S-phase specific.* (Fig. S4A)
- Quantified extranuclear PLA foci. *Result:* *this number is low and similar for all mutants.* (Table S10)

**Reviewer #1:**

Brown, et al. describe the roles of the THO and TRAMP complexes in preventing transcription-replication conflicts (TRCs), DNA breaks, and CAG repeat fragility. When Trf4 and Thp2 are knocked out, CAG repeat fragility, RNA Pol II stalling, and TRCs increase. While increases in R-loops were not observed via DRIP, the authors observed a minor rescue of CAG fragility phenotypes with RNase H overexpression in thp2Δ mutants but not in trf4Δ mutants. The trf4Δ mutants were instead shown to exhibit reduced RPA binding to the CAG repeats, leading to the original observation that CAG fragility and TRC phenotypes in trf4Δ mutants could be rescued with RPA overexpression. Overall, this manuscript is well-written and brings welcome clarifications to the mechanisms by which RNA biogenesis factors prevent CAG repeat instability. A particularly exciting and novel aspect of this manuscript is the observation that RPA overexpression can at least partially rescue the CAG fragility phenotype. This will have exciting implications for evaluating similar RPA depletion in other models of defective RNA processing. The work is also nicely referenced and connects the importance of RNA metabolic processes (e.g., RNA export and degradation) and their role in maintaining genome stability.

General Comments
One of the surprising findings is that authors did not detect R-loop accumulation at the CAG repeats in thp2Δ mutants by DRIP-qPCR. However, they report that RNase H1 oe causes a slight but significant reduction in fragility and a larger reduction in TRC as measured by PCNA-RNAPII PLAs. In their model, the authors propose that stalled RNAPIIs may be the actual drivers of the instability in this mutant but that RNase H1 oe may alleviate in part the phenotype by releasing stalled RNAPIIs. The manuscript would be significantly strengthened if this was tested directly using RNAPII ChIP over the test region with and without RNase H1.

We have now redone the RNAPII ChIP experiment in wild-type, *thp2Δ*, and *trf4Δ* strains with and without RNase H1 overexpression (new Fig. 3E, S2C). We repeated our result that there is increased RNAPII enriched at the CAG repeat tract in *thp2Δ* and *trf4Δ* strains. The new data expressed as IP/Input shows a 4.4-fold increase in the *thp2Δ* mutant and a 2.3-fold increase in the *trf4Δ* strain at the CAG locus compared to wild-type. We compared to two other loci (*MMR1* and *ACT1*), and a negative control not transcribed by RNAPII (*OLI1*) and found that RNAPII was also enriched at the *MMR1* and *ACT1* loci (but not *OLI1*) in both mutants (5 & 9-fold for *thp2Δ* and 5 & 8-fold for *trf4Δ* strains, respectively), therefore the enrichment is not unique to the CAG locus (see new Fig. S2C). We have adjusted our conclusions accordingly.

Interestingly, we observed a 2.5-fold reduction in RNAPII upon RNase H1 overexpression in the *thp2Δ* mutant but not in wild-type and only a mild (non-significant) effect in the *trf4Δ* mutant (see Fig. 3E). This supports our hypothesis that RNase H1 overexpression releases stalled RNAPII in the *thp2Δ* mutant and is consistent with the fact that RNase H1 overexpression decreases CAG fragility in this mutant. It also supports our conclusion that the cause of TRCs in the *trf4Δ* mutants is different and is consistent with our hypothesis that the primary cause of genome instability in TRAMP mutants is RPA depletion.

In that same vein, the authors claim that R-loops "functionally" accumulate in the thp2Δ mutant even though they are not physically detectable. This is an interesting pirouette... I believe that in all fairness, the authors should state that they can't rule out that RNase H1 may not function on co-transcriptional R-loops but instead may possess other functions that ameliorate TRCs. The Pasero lab, for instance, has suggested that RNase H1 may help the restart of stalled forks (ref 75, 76). This disclaimer will be all the more needed if the experiment asked above does not provide the expected results…

We have added in additional explanations for what RNase H1 overexpression may be doing to ameliorate TRCs. Based on newly added RNAPII ChIP results with a RNase H1 overexpression strain (see Fig. 3E and S2C), we now have more support for our proposal that RNase H1 is releasing stalled RNAPII in the *thp2Δ* mutant. See lines 385-388, 436-440: “*Considering the effect of RNase H1 overexpression on both PLA foci and RNAPII ChIP, we propose that overexpression of RNase H1 degrades RNAPII-associated hybrids in the THO mutant, to release stalled RNAPII and resolve TRCs*.” and 554-564: “*Our result that RNAPII stalling is suppressed upon RNase H1 overexpression suggests that digestion of the RNA of a co-transcriptional RNA:DNA hybrid could release stalled RNAPII, leading to the observed suppression of TRCs and breaks*.”

However, our new data does not provide the same level of support that RNase H1 is acting on co-transcriptional R-loops in the *trf4Δ* mutant, strengthening our model that it is likely acting through a different mechanism (perhaps resolving hybrids at the replication fork, as suggested above and in our model). We have now clarified this point in the writing.

The point that RNase H1 may affect replication fork restart is of course, brought to the fore by the findings in the trf4Δ mutant, where RNase H1 oe has a strong effect on TRCs but no effect on fragility. This suggests that the increased fragility in that context is not due to TRCs. In fact, this raises the possibility that the instability in that context may not be due to the transcription through the CAG repeat anymore. The authors assume that the RNA species responsible for titrating RPA originate from rCUG transcripts. I would gather instead that these transcripts represent a small proportion of the transcripts that accumulate genome-wide in this mutant. The manuscript would be significantly strengthened if this was tested directly by blocking / modulating transcription through the CAG repeats.

We agree that the RNA species responsible for titrating RPA likely do not just originate from rCUG transcripts, as RNA levels have been shown to universally increase in TRAMP and exosome mutants (see Houseley et al. EMBO 2007), a point we now make more clearly in the text (lines 340-344 in results and 628-646 in discussion).

We attempted the suggested experiment by blocking transcription of the CAG repeat tract by using terminators placed on either side of the tract (termed 2T-YAC, see Su and Freudenreich *PNAS* 2017 for original use of this YAC) in wild-type, *thp2Δ*, and *trf4Δ* strains, but ran into the issue that the terminators do not fully block transcription, and in fact increased cryptic antisense transcription (especially in *trf4Δ*). Thus, this was not a suitable system to reduce transcription. Therefore, we currently do not have a system to efficiently block transcription or detect TRCs specifically at the CAG locus to fully test this idea. We instead measured strand specific transcript levels at the CAG repeat tract resulting from readthrough from the nearby *URA3* gene and antisense transcription in all 3 strains (new Fig. 3C). We observed ~2-fold more rCAG (antisense) transcripts in the *trf4Δ* mutant than in wild-type, suggesting that cryptic transcripts are increased in this mutant at the CAG repeat like many other RNA transcripts.

However, the main point is that we are not claiming that the rCAG/rCUG transcripts alone are causing the RPA titration effect, so even if we eliminated rCAG and rCUG transcription, we wouldn’t expect to reduce the genome-wide increase in TRCs in the *trf4Δ* mutant (now more clearly stated, see lines 631-633). Our model for how genome instability occurs in the *trf4Δ* mutant (see Fig. 6, right pathway) also predicts that only a portion (if any) of the CAG fragility would be due to TRCs at that locus and is mostly due to defects in fork recovery due to RPA depletion, which is consistent with all our data (now more clearly stated, see lines 613-621).

Specific concerns:
There are general concerns regarding experimental design in several of the figures. For example, the number of data points in different experiments is variable and it is unclear if these are from individual experiments or biological replicates. As per the legends, some experiments appear to only be performed once instead of in biological triplicate (e.g., PLA). Finally, many of the experiments are normalized, but it is unclear what they are normalized to. The wild-type samples are often not "1" (e.g., ChIP). These concerns should be clarified moving forward.

All datapoints shown are individual experiments that are biological replicates, except for the PLA experiments. In most cases, experiments were completed with three biological replicates from at least two isolated strains were used. For the PLA experiments, each data point is number of foci in an individual nucleus and three biological replicates were done for each strain (at least 100 nuclei quantified for each biological replicate; except for nocodazole experiments in which two biological replicates were done (therefore 200 total nuclei were quantified). This is now clarified in the methods (see starting at line 664) and figure legends. For fragility assays, each experiment is actually a rate calculated from 10 colonies (see response below for details on this assay). For the ChIP, all experiments have been redone and the results presented as IP/INPUT (not normalized to the *ACT1* locus) and comparison loci values presented in the supplement.

For DRIP-qPCR/ChIP: could the authors please report the raw % input values instead of normalized values (perhaps in Supp material). This is essential in order to determine the DRIP/ChIP efficiency especially since authors claim to not be able to detect some R-loops.

All DRIP and ChIP experiments were redone, and we now report IP/INPUT values for the CAG locus in the main figures and IP/Input values for comparison loci in the supplement (see Figures 3B, 3E, 5A, S2, and S3C). All raw IP/INPUT values are reported in the supplemental tables (see Tables S6, S9, and S12 for DRIP, RNAPII ChIP and RPA ChIP data, respectively).

Figures (General): Many of the graphs have varying numbers of datapoints. For example, in Figure 1, Wild-type has 3, thp2Δ has 7, trf4Δ has 3, etc. The legend indicates that "each dot represents an individual data point" and "an average of the experiments ± SEM are shown." Are the individual datapoints then an example of one experiment or the combination of experiments?

Additional clarifications about what each of the datapoints in the figures describes have now been included in the methods and figure legends. The individual datapoints are each one independent experiment that is a biological replicate. For all fragility assays, each data point represents one replicate of a 10-colony assay using 10 independent colonies grown in 10 independent cultures (except one-colony assays in Fig. 2A, which were 10 independently grown cultures arising from one colony due to technical limitations). So essentially, 10 independent experiments were used to calculate one value of the most likely rate (and this one value is one data point for the fragility assays). Also, we have tested at least two independent isolates of each mutant strain (except for P*_MET25_*-*RNH1* strains as only one strain was made per mutant). The bar height shows the average value of all experiments done. Our standard is to do at least 3 independent biological replicates per strain or condition, but sometimes we did more if the spread was large, or as we repeated assays for other strains to have a side-by-side comparison, and in this case we showed all the experiments done.

Figure 2: With the revised one-colony fragility assay, are wild-type cells still able to be used as a negative control? If so, these data should include the wild-type FOAR data for both A and C.

We have assayed the wild-type strain in the same way and have now included this wild-type data as a negative control for the one-colony fragility assay (see Fig. 2A and Table S1B). This data follows the same trend as a normal 10-colony fragility assay in which wild-type has the lowest FOA-resistance rate and it is increased in the *thp2Δ*, and *trf4Δ* strains.

Figure 4 - How many biological replicates of PLA were included in the study? From the Figure legend, it appears that only 1 replicate was performed.

Three biological replicates were used for each of the PLA experiments included in the study (except for the nocodazole treatment; 2 were performed for 200 total nuclei screened per strain). At least 100 nuclei were screened per replicate for a total of at least 300 nuclei screened for each strain/condition.

Minor comments:
Line 283: Replace the word "massive".

This change was made and is in red text, new line 272.

Line 305-306: "We previously showed that R-loops form preferentially at expanded CAG tracts in vivo and that increasing R-loop stability by removing both RNase H1 and RNase H2". I'm not sure the word "preferentially" should be used. The previous study showed a slight increased R-loops loads at the CAG tract, this does not make it "preferential". Similarly, I do not believe that the authors measured "R-loop stability" per se (half-life) in their previous work. This should be rephrased using more neutral language.

This change was made and is in red text, new lines 289-291.

Line 315: the adjective "stable" is not necessary.

This adjective was removed, new line 300.

Line 520-522: The suggestion that DRIP may not recognize short R-loops should not be oversold. Rewrite to "It has been recently SUGGESTED that small R-loops (60 bp or less) associated with paused RNAPII MAY not BE WELL detected by DRIP, which preferentially detects longer (>200 bp), more stable R-loops [55].

This change was made and is in red text, new lines 543-547.

**Reviewer #2:**

(David Tollervey, signs review): The authors investigate the basis of CAG repeat instability using a model system in yeast, and find that loss of the RNA BP Thp2 and the TRAMP component Trf4 synergistically enhance repeat DNA fragility. Interestingly, this is attributed to different defects in the single mutants; R-loop formation in thp2∆ and TRCs due to RPA depletion in trf4∆.

The results are clearly presented and will be of wide interest. I would support publication with only minor changes.

Minor points:

1: The authors might also consider the possibility that the exosome/TRAMP complexes might directly prevent and/or resolve R-loops by degradation of the RNA strand. A potential increase in RNA/DNA hybrid formation in absence of Trf4 lead to reduced binding of RPA to the single stranded DNA. TRCs in the single trf4 mutant were compensated by overexpression of RNH1.

Thanks for pointing out this alternative hypothesis, which we have now added to our discussion (lines 640-642). This would be consistent with data from other labs that showed that removal of RNA:DNA hybrids was needed to restore RPA binding, though these hybrids would either need to be poorly detected by DRIP or at some subset of sites.

2: The "DRIP" technique reported by the Chedin lab relied on naked DNA as starting material. The authors used antibody S9.6, but performed a variant of DRIP technology that relies on cross-linked DNA. It is possible that formaldehyde crosslinking could shield R-loops, thereby reducing the efficiency of their detection?

This is a good point. The DRIP experiments were repeated without crosslinking and sonication (restriction enzyme digestion was used instead to fragment DNA). We followed the protocol cited in Garcia-Rubio et al. *Methods Mol. Bio.* 2018 (see Materials and Methods lines 703-719 for further details). We obtained the same results as before but have replaced the old data with the new data since it is the preferred method in the field (see new Fig. 3B, S2A, S2B).

3: P19 and Fig. 4B: The authors could usefully describe data in panel C (RNH-/+) in this paragraph and not only in the Discussion. They might also add a small interpretation/conclusion, as to why over-expression of RNase H1 decreases the PLA signal in single thp2 delta and trf4 delta mutants.

We have added an additional explanation of this data to the results section (lines 436-440). Based on our new ChIP results with RNase H1 overexpression, we propose that RNase H1 is releasing stalled RNAPII in the *thp2Δ* mutant, but is likely acting through a different mechanism in the *trf4Δ* mutant (for example degrading RNA:DNA hybrids at stalled replication forks or breaks).

4: It is unclear how the authors validated the efficiency of the S9.6 antibody. A control with/without in vitro treatment with recombinant RNase H might have been useful to assess the specificity of antibody S9.6.

The DRIP experiments were repeated (see point 2 for new method) and half the samples were treated in vitro with RNase H to determine which portion of the signal was due to RNA:DNA hybrids. The new data are shown in Fig. 3B, S2A, S2B.

5: As a comment, it is not clear that R-loops at the CAG repeats should be short (60 bp or less)? In reference 55 the authors suggested that R-loop accumulation at RNAPII promoter-proximal pause sites could be disrupted by the DRIP. This is understandable as nascent RNAs at promoter-proximal pausing sites are shorts, so R-loops should be inherently small. However, in the body of the gene, pausing of RNAP II might lead to formation of longer R-loops that would be detected by DRIP.

Agreed. We softened our language and invoked the possibilities of stability or association with RNAPII as well as length. Based on our new data showing RNAPII release upon RNaseH1 overexpression in the *thp2Δ* mutant, we used the following phrasing “we hypothesize that they [R-loops] are transient and likely associated with the stalled RNAPII, rather than stable R-loops left after passage of the transcriptional machinery” (lines 542-543) and “We conclude that R-loops within the CAG tract in the THO-defective mutant are different than those that accumulate in the *rnh1∆rnh201∆* mutant and may be shorter or more transient since they were not detected by DRIP [60] but do contribute to causing the increase in CAG fragility observed in this background” (Lines 353-356). It is also possible that short hybrids still associated with RNAPII are shielded from detection by the S9.6 antibody (possibility added to the discussion, lines 545-547).

6: Line 161: Did the authors mean in absence of both RNase H1 and RNase H2?

We meant in the absence of both RNase H1 and H2. This change was made and is in red text (lines 163-164).

7: P27: There is no mention of Trf4 in REF 72

Ref 72 was removed and replaced with an appropriate reference (line 603).

**Reviewer #3:**

In this article the authors study the impact of the THO complex and the TRAMP subunit Trf4 in maintaining CAG repeat stability and in the more general prevention of transcription replication conflicts. They show that deletion of the Thp2 and Mft1 members of the THO and of Trf4 and Rrp6, both involved in degradation of RNA in the nucleus, induces instability in a repeat-containing YAC. The mechanisms proposed for this instability are likely different, only the first seemingly involving increased formation of R-loops, at least as demonstrated by the suppression of the phenotype by RNaseH1 overexpression. Based on the increased RNAPII occupancy detected by ChIP in the region of the repeats the authors propose that excess transcription-replication conflicts might occur in mutants. Seemingly in agreement with this hypothesis, increased Proximity Ligation Assay signals generated by PCNA and RNAPII are detected in nuclei of mutant cells, which are suppressed by overexpression of RNaseH1 (but both in thp2∆ and trf4∆ cells, see below). The authors propose that instability in trf4∆ cells results from the titration of RPA by excess undigested RNA. Consistently, overexpression of RPA suppresses both the repeat-induced fragility and the increased PLA foci in trf4∆ cells.

This is a potentially interesting study, but not all of the conclusions are adequately supported and many important controls are missing.  A few overstatements should be corrected or further supported. The possible titration of RPA by excess RNA is particularly interesting. A positive recommendation would certainly require providing the missing controls and addressing the concerns below.

Major points:

Fig. 1 and lines 203-207: The rate of appearance of 5-FOA resistant colonies in thp2∆ cells is 4 times that of wt cells in the absence of the CAG repeat and 4.7 in the presence of the repeat. According to this result the thp2∆ mutation affects equally the loss of the distal URA3 gene in the presence or the absence of the repeat. There is no significant exacerbation of the phenotype in the presence of the repeats as the authors say, which raises a serious concern about the interpretation of the results.

We agree and have removed the claim that fragility is exacerbated by the CAG tract in this mutant “indicating that the *thp2∆* fragility phenotype is not unique to expanded triplet repeats” (line 209; see also lines 235-238 for a similar statement about the trf4 mutant). Even though we initially found the effect using a CAG tract fragility assay, it is also evident from our TRC and ChIP data that both *thp2Δ*, and *trf4Δ* mutants have a genome-wide effect, and we have gone through the paper to make sure that is more clearly stated. Some examples include:

“A similar pattern was observed at two gene loci, the highly transcribed *ACT1* locus and the lower transcribed *MMR1* locus, with 5-9-fold increases in RNAPII occupancy in *thp2∆* and 5-8-fold increase in *trf4∆* strains, indicating that RNAPII accumulation on chromatin may be universally increased in these mutants (Fig. S2C).” (See lines 371-374)

“RPA levels were also reduced by 3.1-fold at the *ACT1* locus in *trf4∆* cells (Fig. 5A and S3C), indicating that there is a global deficiency in RPA association with DNA in this background.” (See lines 458-460)

“Since overexpression of RNase H1 decreased RNAPII stalling at the CAG repeat tract and TRCs genome-wide in the *thp2∆* mutant, these tethered RNA polymerases likely block the replication fork, leading to TRCs, fragility, and repeat contractions.” (See lines 551-553)

Fig. 3B: in the DRIP experiment the RNaseH treatment is missing. This is absolutely required for demonstrating that the DRIP signal is generated by R-loops. Also it is not clear why the data are normalized to MMR1: is this a region deprived of R-Loops? MMR1 forms R-loops according to the data from last Aguilera's paper (San Martin-Alonso et al.). The authors should justify the use of such a reference and repeat all DRIP experiments by including an RNaseH treated control which is a commonly used standard

The DRIP experiments were repeated with a new protocol without crosslinking or sonication and including the RNase H treatment control. We followed the protocol cited in Garcia-Rubio et al. *Methods Mol. Bio.* 2018 (see Materials and Methods for further details). For the new data we did not normalize to any gene loci and instead reported only IP/INPUT values and showed the DRIP signal at several loci for comparison (Fig. 3B and S2A). The conclusions did not change.

Fig. 3C: Inferring from suppression by RNaseH overexpression that R-loops form but are not detected at the repeats is rather speculative. Can the authors include a positive control showing they can actually see DRIP signals in THO mutants?

We included *PMA1* as a locus that showed increased R-loops in a *thp2∆* mutant in Pfeiffer et al. *EMBO* 2013. We observed a mild increase in DRIP signal above wild-type that was reduced upon RNase H treatment. There was also a large increase in the DRIP signal at the *PMA1* locus in the *rnh1∆rnh201∆* mutant, confirming that we can detect R-loops, at least the type that occur in this background (new Fig. S2A). Our new data showing RNAPII removal at the CAG repeat upon RNase H1 overexpression supports that a RNAPII-associated hybrid is likely present, even if not detected by DRIP. We think these results are important for the field; that not all functional hybrids are easily detected by DRIP (maybe RNAPII-associated hybrids are shielded by the protein?).

Fig. 3D: The RNAPII ChIP indicates increased polymerase occupancy but does not allow establishing if the signal represent stalled polymerases or increased transcription in the region (i.e. due to increased initiation). The authors should either prove there is no increased initiation or be more cautious in the interpretation of this result. Also, normalizing to ACT1 assumes there is no change for this gene. If this is the case, it should be shown. Also, the ChIP signals should be shown relative to a negative control (e.g.  region with no polymerase).

We have now measured strand specific rCAG and rCUG transcript levels at the CAG locus (new Fig. 3C). We observed no increase in transcript levels compared to wild-type in the *thp2∆* strain (if anything a decrease), supporting that the observed RNAPII enrichment at the CAG repeat in that strain is likely due to RNAPII stalling. However, we did see a slight increase in transcripts in the *trf4∆* (2.1 and 1.3-fold over wild-type for rCAG and rCUG transcripts, respectively), though we think this could also be due to lack of transcript processing (rather than increased transcription) in *trf4∆*. Our new RNAPII ChIP data show a somewhat higher level of RNAPII over the CAG tract in *thp2* (4.4-fold), than in *trf4* (2.3-fold) strains. Together, these results led us to be more cautious with our conclusions about RNAPII stalling in this mutant (“There is a possibility that RNAPII enrichment may be due to either increased transcription through a genomic region or RNAPII stalling. Since there is no significant increase in rCUG/rCAG transcript levels in the *thp2∆* mutant compared to wild-type (Fig. 3C; Table S9), RNAPII enrichment is likely due to RNAPII stalling in that mutant. However, we cannot rule out a transcription effect in the *trf4∆* mutant, as there is a 2.1-fold increase in rCAG and 1.3-fold increase in rCUG transcripts compared to wild-type (Fig. 3C; Table S7), though this could also be due to accumulation of unprocessed transcripts rather than increased transcription.” – See lines 375-381)

We reported all new RNAPII ChIP data as IP/INPUT not normalized to *ACT1* and show *ACT1* levels in the supplement. Indeed, RNAPII levels are also increased 8-9-fold at *ACT1* (and 5-fold *MMR1* a lowly transcribed gene) (see Fig. S2C), indicating that the increased RNAPII occupancy in both mutants is not specific to the repeat, consistent with the genome-wide TRCs and generally increased fragility we observed. We have changed our wording to point out that the effect is not specific to the CAG tract (lines 371-374).

We have now included *OLI1* as a negative locus, as it is in the mitochondrial genome and is not transcribed by RNAPII (see Fig. S2C). We observe no enrichment of RNAPII at *OLI1*. As an additional positive control, we include data using a P_GAL1_-CAG-100 YAC that drives transcription through the CAG repeat when grown in galactose but not glucose (see Fig. S2C). RNAPII enrichment was greatly increased in the galactose condition compared to glucose at the CAG repeat.

Fig. 4A. PLA experiments are subject to artifacts. The authors should provide controls showing that no foci are seen in the absence of replication (e.g. in G1 arrested cells) or transcription (use of transcription inhibitors). Also, they should systematically count for each mutant the number of foci that are extranuclear, to evaluate the reliability of the technique.

We have now added this control. Cells were treated with nocodazole (to arrest in M phase) and that reduced the amount of PLA foci/TRCs detected in wild-type, *thp2∆*, *rnh1∆rnh201∆*, and *trf4∆* strains to almost zero (see new Fig. S4A). Extranuclear foci were counted for all strains/conditions and are summarized in Table S10. There was no difference between strains, hovering around 20% on average. Nocodazole treatment did increase the percent of extranuclear foci, suggesting that most are not relate to S phase TRCs.

Fig 4BC. If trf4∆ induces genomic instability independently of R-loops, why overexpression of RNaseH1 suppresses formation of PLA foci in these cells? This result does not appear to fit their model, according to what claimed in p.17 (lines 368-369). Also, it appears incoherent with the fact that overexpression of RNase H1 does not suppress instability in trf4∆ cells (Fig 3D).

Since RNase H1 overexpression suppresses PLA foci but not CAG fragility (and minimally RNAPII occupancy at the CAG tract (new ChIP data, Fig. 3E), this is most consistent with CAG fragility occurring upstream of TRCs in this mutant. Since CAG fragility is dependent on RPA levels, we hypothesize that the primary defect in this mutant is RPA deficiency at stalled replication forks, and this is what leads to fragility at the CAG tract (which is naturally a fork stalling element). This is why we have placed a fragility pathway upstream of TRCs in our model in the TRAMP mutant (see TRAMP4 side of Fig. 6, now labeled fragility pathway 1). Our model is that both TRCs and the RNA:DNA hybrids targeted by RNaseH1 overexpression are a late event, but that their degradation is important for resolution of TRCs (and perhaps also prevention of fragility; now labeled pathway 2 on the TRAMP4 side of Fig. 6).

It is quite possible that the balance of these pathways is somewhat different at the CAG tract (where we are measuring fragility, RNA:DNA hybrids, and RNAPII stalling) and other areas of the genome (which are being measured in the PLA assay). Because the CAG tract is a natural site of fork stalling and forms DNA structures, it could be particularly sensitive to the RPA depletion step and break more often using the fragility pathway 1. In contrast, highly transcribed gene bodies could be more sensitive to the fragility pathway 2 (now added to the Fig. 6 model legend).

We have now improved our discussion of our model, see lines 615-620, which we hope clarifies it. We emphasize that it is just a model at this point, which fits our data, but it will need further testing which is beyond the scope of this paper. Nonetheless, we hope our unexpected results regarding the importance of RPA levels in protecting against both CAG fragility and genome-wide TRCs in TRAMP4 mutants stimulates the field to consider new models for why genome instability occurs in this background.

Fig 5A RPA ChIP should be controlled using a negative control (e.g. no antibody) and by showing (rather than normalizing to) the levels at ACT1. According to the model proposed, RPA should be sequestered by excess RNA in the trf4∆ context. If so, binding of RPA should be generally affected, and normalization to ACT1 would not be acceptable. If binding of RPA to ACT1 is not affected, the authors should explain why.

RPA ChIP was repeated in wild-type and *trf4∆* strains with/without RPA overexpression (see Fig. 5A, S3C, and Table S12). We now report IP/INPUT values and did not normalize to *ACT1* but show it alongside, as suggested. We also included samples performing the immunoprecipitation with IgG as a negative control (see Fig. S3C). The new experiments confirm that there is more RPA binding at the CAG locus than the *ACT1* locus, and also that RPA binding is significantly reduced in the *trf4∆* strain at both loci (5.2-fold at CAG-70, 3.1-fold at *ACT1*). We have adjusted our conclusions from this experiment accordingly (see lines 458-460).

Fig5B The authors should show that overexpression of RPA leads to increased levels of the protein and that its recruitment to CAG-70 is restored in these conditions.

We have now done western blots and confirmed that there is increased Rfa1, Rfa2 and Rfa3 protein levels upon RPA overexpression (see Fig. S3B and Table S14). Blot quantification shows a 2.7-10-fold increase in Rfa1/Rfa2 protein levels and 15-19-fold increase in Rfa3 protein levels. This is less than the 20-40x increase shown by our previous RT-PCR experiments to detect transcripts but may also be subject to being in a non-linear quantification range. In any case, all 3 subunits show increased protein levels.

We also did RPA ChIP in wild-type and *trf4∆* strains with and without RPA overexpression (see Fig. 5A and S3C). There was a mild rescue of RPA detected at the CAG repeat in the *trf4∆* strain upon RPA overexpression, though only a 1.7-fold increase. This was less than we expected, but perhaps the additional RPA available is not able to bind to the genome very effectively, since it is still a mutant background with increased dsRNA levels.
